# Scoping review of anticancer drug utilization in lung cancer patients at the end of life

**DOI:** 10.1007/s12094-024-03711-1

**Published:** 2024-10-05

**Authors:** Endre Szigethy, Mohammed Merzah, Ivan Sola, Gerard Urrútia, Xavier Bonfill

**Affiliations:** 1 PhD Programme in Biomedical Research Methodology and Public Health, Universitat Autònoma de Barcelona, Barcelona, Spain; 2 Epidy Kft, Csúcs Utca 9, Debrecen, 4034 Hungary; 3 Department of Public Health and Epidemiology, Faculty of Medicine, University of Debrecen, Debrecen, Hungary; 4 Technical Institute of Karbala, Al Furat Al Awsat Technical University, Kufa, Iraq; 5 Iberoamerican Cochrane Centre, Biomedical Research Institute Sant Pau (IIB Sant Pau), CIBER Epidemiología y Salud Pública (CIBERESP), Universitat Autònoma de Barcelona, Barcelona, Spain

**Keywords:** Lung cancer, Anticancer agents, Terminal care, Medical overuse

## Abstract

**Purpose:**

This scoping review aims to deepen the understanding of end-of-life anticancer drug use in lung cancer patients, a disease marked by high mortality and symptom burden. Insight into unique end-of-life treatment patterns is crucial for improving the appropriateness of cancer care for these patients.

**Methods:**

Comprehensive searches were carried out in Medline and Embase to find articles on the utilization of anticancer drugs in the end of life of lung cancer patients.

**Results:**

We identified 68 publications, highlighting the methodological characteristics of studies including the timing of the research, disease condition, treatment regimen, type of treatment, and features of the treatment. We outlined the frequency of anticancer drug use throughout different end-of-life periods.

**Conclusion:**

This review provides a comprehensive overview of primary studies exploring end-of-life treatments in lung cancer patients. Methodological inconsistencies pose many challenges, revealing a notable proportion of patients experiencing potential overtreatment, warranting more standardized research methods for robust evaluations.

## Introduction

The application of anticancer drugs during end-of-life care represents a complex and debated issue, with significant variability observed in their utilization across diverse methodological contexts [1–3]. The administration of anticancer drugs near the end-of-the-life period is progressively prevalent, especially among patients with solid tumors such as lung, breast, and colorectal cancer [1, 4–9].

The real merits of these treatments in terms of survival enhancement and life quality improvement are often ambiguous, and in certain instances, they may be overshadowed by potential harms, including adverse reactions and treatment burdens [1, 7, 10–12]. An expanding corpus of literature posits that the decision to employ anticancer drugs during the end-of-life period is shaped by a broad spectrum of factors, such as patient preferences, physician’s attitudes, healthcare system characteristics [13–16], or cancer types [17].

The substantial financial burden of these therapies raises ethical and economic concerns, leading to increased advocacy for palliative care, which prioritizes comfort and quality of life (QoL) over curative efforts [2, 12, 18]. Palliative care, emphasizing symptom management and psychological support, is increasingly recognized as a more suitable approach for end of life, focusing on dignity, patient preferences, and enhancing QoL rather than merely prolonging life. This shift reflects the growing understanding that QoL is a crucial measure of effective end-of-life care [19, 20].

In discussing the use of anticancer drugs during end-of-life care, it is important to define what is meant by “end-of-life” In this context, the term refers to the final days, weeks, or months of a person’s life, during which it becomes medically evident that death is imminent, and a terminal moribund state cannot be prevented [7].

Our recent scoping review on end-of-life anticancer treatment analyzes the methodologies and outcomes related to the publications on anticancer drugs during the end of life [21]. Fundamental methodological attributes of these publications were outlined, particularly in terms of determining treatment frequency, thus underscoring the challenges associated with direct comparisons among clinical studies. These parameters include, but are not limited to, the timing of research, disease status, definition of treatment regimen, type of therapy, and treatment attributes. Approximately half of the studies described the frequency of end-of-life anticancer drug administration in patients with any cancer type, encompassing all malignancy forms. The overall frequency of end-of-life anticancer drug administration demonstrated a broad range across all end-of-life periods, influenced by factors such as data source, design, study population size, disease status, treatment characteristics, treatment type, and region. Due to the different nature of the specific tumor types, specific analyses by cancer site surpassed the boundaries intended for that scoping review article.

Nonetheless, the exploration of end-of-life cancer treatment based on specific cancer types is of critical importance in oncology and palliative treatment. This is due to the mentioned variability in the application and intensity of end-of-life care across diverse cancer tumors, leading to potential imbalances in care delivery [1, 4–7, 14, 22]. Decedents from lung cancer, for instance, often have a heavier symptom burden than other cancers, and a rapid decline toward the end of life [23, 24]. Given the distinct characteristics of lung cancer, tailored therapeutic strategies are essential. By focusing specifically on lung cancer, studies can yield more precise and relevant insights than those addressing overall cancer types. This targeted approach enables a more effective enhancement of end-of-life care by directly addressing the unique needs of lung cancer patients, which a broader, multi-cancer study might overlook. Since lung cancer is a prevalent malignant disease responsible for the highest mortality rate among male cancer patients in numerous countries [25–28], a comprehensive understanding of its unique treatment patterns is a significant aspect of cancer care. Therefore, the objective of our study was to establish a comprehensive understanding of the patterns and utilization of anticancer treatment during the end-of-life period in lung cancer patients.

## Methods

### Study identification

The review’s methodology, as detailed in our preceding publication, was streamlined using a scoping review approach suggested by JBI PRIMSA ScR [29–32]. A systematic search was conducted across Medline (via PubMed) and Embase (via Embase) literature databases, using keyword combinations such as ‘lung/bronchial/mesothelioma/pulmonary/thoracic’, ‘aggressive’, end-of-life’, ‘last days/weeks/months’, ‘until/near death’, ‘late/end stage’, ‘days/weeks/months of life’, ‘antineoplastic’, ‘anticancer’, ‘chemotherapy’, ‘hormone/immune/biological/targeted’, and ‘cancer/malignancy/neoplasm/tumour and care/drug/treatment/therapy’. The search strategy developed for Medline is displayed in Appendix 1. The research protocol is available upon request.

### Eligibility criteria

An initial exhaustive literature search for the diverse cancer types was executed in November 2020, yielding a total of 13,476 articles. This search was subsequently updated on August 22, 2023, adding 1129 publications. After the elimination of duplicates, we evaluated 13,789 references based on their titles and abstracts. A total of 68 publications were selected for an in-depth analysis.

### Selection of studies

Two authors (ES and MM) assessed titles and abstracts against the eligibility criteria and potentially relevant full-text articles were selected. Discrepancies across reviewers were resolved through discussion or by consensus. Based on linguistic proficiency of authors, articles were accepted if published in English, Spanish, French, German, Hungarian, or Arabic.

### Data extraction

Two independent reviewers collated data from each paper, reconciling any discrepancies through consensus, about the study duration, research design, data origin, geographical location, patient demographics (number of patients, age, and gender), cancer stage, type of lung cancer, stipulated inclusion and exclusion criteria, type and classification of treatment (as defined in the individual articles), reported end-of-life periods, and proportion of patients undergoing anticancer treatment within those periods.

### Analysis and reporting

This scoping review adheres to the guidelines delineated by the Preferred Reporting Items for Systematic Reviews and Meta-Analyses extension for Scoping Reviews (PRISMA-ScR) parameters [33]. The PRISMA-ScR checklist was followed to guarantee the comprehensiveness and transparency of the review methodology and reporting. The publications were grouped based on their methodological characteristics, including study design (prospective or retrospective), data source (medical records or population data, encompassing registries, claims data, and administrative datasets), disease status of the patient cohort (encompassing all cancer stages or advanced cancer patients), patient population definition (if the denominator of the rate includes either all cancer patients or only those previously treated), treatment initiation (commencement of a new anticancer regimen or receiving any form of anticancer treatment), and the type of anticancer treatment (as defined by the individual studies, encompassing biologics, chemotherapy, hormonal/endocrine therapy, immunotherapy, targeted therapy, and systemic anticancer treatment). When reporting multiple end-of-life treatment periods in the same article, these were not considered mutually exclusive but rather inclusive categories, unless otherwise defined in the given article. In such cases, we combined mutually exclusive categories to make the values comparable with other studies. Therefore, in the interpretation of results, it is essential to note that the count of estimates may surpass the number of articles. In such instances, we provide explicit clarification, indicating whether the discussion pertains to the distribution among individual publications or the distribution of estimates. As in any scoping review, the analysis primarily involves a narrative description and reporting frequencies of the extracted data items.

## Results

### Studies identified for review

**Fig. 1:**
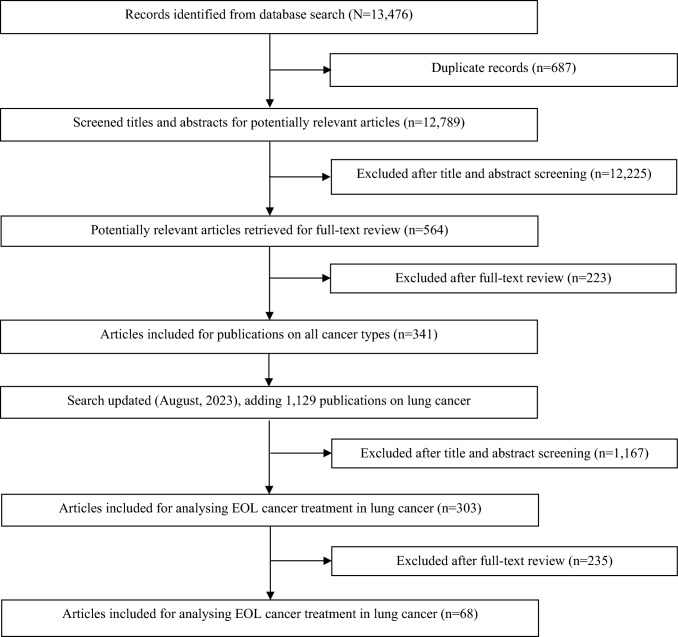
Scoping review flowchart

As part of our previously published scoping review on all cancer types, the first comprehensive search was completed in October 2018. In August 2023, the search was updated with focusing on studies on lung cancer only, resulting in a total of 14,605 articles. After removing duplicates, 13,918 publications were screened for eligibility by titles and abstracts. Subsequently, 68 publications were found reporting relevant lung cancer specific data on the frequency of anticancer drug use at the end of life (Fig. 1). Although the majority were full articles (69.1%), conference abstracts were a non-negligible number (30.9%) (Table 1). The reference list of these publications is displayed in Appendix 2.

**Table 1:** Characteristics of articles describing end-of-life anticancer drug use in lung cancer patients (*N* = 68)

	Distribution of articles (*n*)
Article type	
Abstract	30.9% (21)
Full-text article	69.1% (47)
Publication date	
2009 and before	4.4% (3)
2010–2014	26.5% (18)
2015–2019	47.1% (32)
2020–2023	22.1% (15)
Region	
Asia	16.2% (11)
Austral–Pacific	11.8% (8)
Europe	29.4% (20)
North America	42.6% (29)
Study setting	
Population based	54.4% (37)
Hospital based	45.6% (31)
Disease status	
All cancer stages	44.1% (30)
Advanced stages	55.9% (38)
Treatment characteristics	
Treated and non-treated patients	35.3% (24)
Treated patients	64.7% (44)
Study design	
Prospective	33.8% (23)
Retrospective	66.2% (45)
Study sample (patients)	
<500	45.6% (31)
500–2000	8.8% (6)
>2000	44.1% (30)
Treatment initiation^a^	
Ongoing anticancer treatment	98.5% (67)
Starting new anticancer regimen	1.5% (1)
Ongoing anticancer treatment and starting new anticancer regimen	13.2% (9)
Treatment type^a^	
Anticancer therapy	10.3% (7)
Biological therapy	1.5% (1)
Chemotherapy	80.9% (55)
Immunotherapy	4.4% (3)
Systemic anticancer treatment	10.3% (7)
Targeted therapy	5.9% (4)
Cancer type	
All types of lung cancer	48.5% (33)
Malignant pleural mesothelioma	2.9% (2)
NSCLC	47.1% (32)
Thoracic cancer	1.5% (1)

*n* number of articles, *NSCLC* non-small cell lung cancer

^a^Overlap between categories is possible

### Characteristics and methodological features of selected publications

The chronological overview of publications concerning end-of-life treatment in lung cancer patients revealed notable trends. Before 2009, there was a consistently low publication rate. An increase was observed in 2010, when the number of publications begun to rise steadily, reaching its peak in 2018 with a total of ten papers. While there was a subsequent fluctuation in the following years, the interest remained relatively consistent, as evidenced by the sustained number of publications from 2019 to 2023 (Table 1). In the identified articles, the period of data collection encompassed the span from 1991 to 2020. North America stands out with the highest percentage of publications at 42.6%, followed by Europe with 29.4%, in contrast to other regions. No similar publication was identified in Latin America or Africa (Table 1). The geographical distribution of the studies showed that while the European studies investigated mainly the final 4 weeks before death, studies from North America focused mainly at 2 weeks. Other regions also assessed mainly 1 month. The other indicator of overtreatment, defined as the initiation of a new anticancer regimen in the final month of life, was infrequently documented in other regions different from North America.

**Fig. 2:**
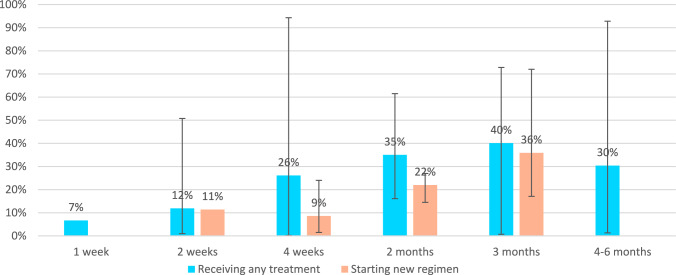
Reported frequencies (mean and range) of anticancer drug use at the end of life in patients with lung cancer (no data was available for the initiation of a new regimen in the last 1 week or during the 4–6 months period. Where no range bar is displayed, only a single data point was identified)

Regarding the study setting, population-based studies slightly predominated over hospital-based studies (54.4% vs. 45.6%, respectively). Studies mainly included lung cancer patients of all stages (55.9%), with a lesser focus on patients specifically in advanced stages (44.1%). The majority of studies encompassed patients with all forms of lung cancer (48.5%), with a similar proportion of researches focusing exclusively on non-small cell lung cancer (NSCLC) (47.1%). A third of these studies did not restrict enrollment based on prior treatment status, whereas the majority (64.7%) enrolled only patients who had already received cancer treatment. Studies including only treated patients were more frequent at both 2 and 4 weeks before death time points compared to those with a mixed population, and especially those assessing starting of a new regimen. The frequency of anticancer drug use at end of life, both in the final 2 and 4 weeks, was evenly distributed in studies focusing on advanced cancer patients and those in all cancer stages. Most research used a retrospective design (66.2%), with a comparable proportion of studies with a sample between less than 500 subjects (45.6%) and those with more than 2000 (44.1%). Research involving smaller cohorts (less than 500 patients) exhibited the broadest spectrum of results throughout all periods of end of life. Among the studies that assessed the treatment rate at 2 weeks before death, studies with >2000 patients predominated, while those with <500 patients predominated among those that assessed the 1-month period. Almost all publications (98.5%) reported ongoing anticancer therapies. The initiation of new treatment regimen across various end-of-life periods for lung cancer patients was reported in ten studies with 42 distinct estimates, with the time frames extending from 2 weeks to 3 months, while only 1.5% were focused solely on the initiation of new regimens, without reporting ongoing anticancer therapies. Chemotherapy constituted the predominant treatment modality assessed (80.9%), while other therapies such as targeted therapy or immunotherapy were reported infrequently (Table 1; Fig. 2).

### Frequency of end-of-life anticancer drug use

From the individual 68 publications, a total of 189 distinct end-of-life anticancer treatment estimates were extracted. The definition of end-of-life periods in the studies fluctuated between a week to half a year. By far, the most reported end-of-life treatment periods were 2 and 4 weeks before death (around 40% of the total estimates each). Few studies evaluated longer periods up to 4–6 months and a single study evaluated 1 week before death. As might be expected, the average treatment rate increased with increasing time, from 6.7% at 1 week to 30.4% at 4–6 months. The mean rates observed at 2 and 4 weeks before death were 11.9% and 26.1%, respectively. It is noteworthy that the ranges observed for all treatment rate estimates were very wide. The 4-week period before death was the most reported end-of-life period for the initiation of new treatment regimen, with a mean rate of 8.6%. The frequency increased with time, with a mean rate of patients initiating a new treatment 3 months before death of 35.9% (Fig. 2).

The stratified analysis of end-of-life anticancer drug utilization focuses on the most frequently studied time frames: the last 2 and 4 weeks before death for ongoing cancer treatment, and the last 4 weeks for the initiation of new treatments, accounting for 83% of all treatment rate estimates. These treatment frequencies are stratified below based on factors such as regional distribution, study design and size, treatment characteristics, and disease attributes (Table 2).

**Table 2:** Reported frequency of anticancer drug use at the end of life in patients with lung cancer, stratified results

	Receiving any treatment, mean (range, *n*)		Starting new regimen, mean (range, *n*)
	2 weeks	1-month	1 month
Region			
Asia	17.9% (14.5–22.1%, *n* = 3)	44.1% (10.1–94.3%, *n* = 14)	15.8% (11.0–19.9%, *n* = 3)
Austral–Pacific	18.3% (1.0–50.8%, *n* = 3)	7.0% (0.5–15.0%, *n* = 9)	–
Europe	12.3% (3.4–26.0%, *n* = 6)	23.3% (0.2–72.0%, *n* = 26)	11.5% (7.0–15.9%, *n* = 4)
North America	11.0% (3.1–42.0%, *n* = 48)	25.7% (8.0–44.0%, *n* = 14)	7.3% (1.5–24.0%, *n* = 27)
Study design			
Prospective	15.9% (1.0–50.8%, *n* = 12)	28.3% (0.6–94.3%, *n* = 19)	9.8% (8.7–10.8%, *n* = 2)
Retrospective	10.9% (3.1–29.0%, *n* = 48)	25.2% (0.2–72.0%, *n* = 44)	8.5% (1.5–24.0%, *n* = 32)
Study sample (patients)			
<500	18.8% (3.0–50.8%, *n* = 15)	25.5% (0.5–94.3%, *n* = 32)	14.1% (5.0–24.0%, *n* = 8)
500–2000	10.2% (3.1–14.5%, *n* = 3)	33.0% (11.0–72.0%, *n* = 8)	9.2% (3.0–13.0%, *n* = 5)
>2000	9.1% (1.0–18.6%, *n* = 41)	24.5% (0.2–56.0%, *n* = 23)	6.3% (1.5–19.9%, *n* = 21)
Study setting			
Hospital based	20.1% (3.0–50.8%, *n* = 11)	27.5% (0.5–94.3%, *n* = 34)	12.6% (7.0–17.0%, *n* = 7)
Population based	10.0% (1.0–42.0%, *n* = 49)	24.5% (0.2–60.7%, *n* = 29)	7.5% (1.5–24.0%, *n* = 27)
Disease status			
Advanced stages	11.4% (4.0–42.0%, *n* = 21)	29.3% (7.0–56.7%, *n* = 23)	14.0% (8.7–19.9%, *n* = 6)
All cancer stages	12.1% (1.0–50.8%, *n* = 39)	24.3% (0.2–94.3%, *n* = 40)	7.4% (1.5–24.0%, *n* = 28)
Treatment characteristics			
Treated and non-treated patients	13.8% (4.0–42.0%, *n* = 8)	21.7% (0.5–60.7%, *n* = 24)	7.0% (*n* = 1)
Treated patients	11.6% (1.0–50.8%, *n* = 52)	28.9% (0.2–94.3%, *n* = 39)	8.6% (1.5–24.0%, *n* = 33)
Treatment type			
Anticancer therapy	3.4% (*n* = 1)	25.1% (8.4–44.0%, *n* = 6)	10.8% (*n* = 1)
Biological therapy	–	0.2% (*n* = 1)	–
Chemotherapy	11.5% (1.0–42.0%, *n* = 56)	28.9% (5.4–94.3%, *n* = 42)	8.1% (1.5–24.0%, *n* = 28)
Immunotherapy	–	23.2% (7.3–44.5%, *n* = 3)	12.3% (8.7–15.9%, *n* = 2)
Systemic anticancer treatment	21.7% (6.7–50.8%, *n* = 3)	13.5% (0.6–27.0%, *n* = 6)	–
Targeted therapy	–	26.2% (0.5–72.0%, *n* = 5)	10.0% (7.0–12.0%, *n* = 3)

*n* number of estimates

The differences observed in treatment rates by region are striking, being similar in Europe and North America (approximately 12% at 2 weeks and 24% at 4 weeks before death). Asian rates are much higher in both periods, while Austral–Pacific rates were discrepant.

Prospective studies primarily concentrated on the final month of life, displaying a broad spectrum of anticancer drug utilization and a mean treatment rate of 28.3%. Retrospective studies, on the other hand, more frequently reported treatment frequencies during the last 2 weeks of life (mean treatment rate of 10.9% compared to 25.2% at 1 month before death). The initiation of new anticancer treatment regimen was infrequently documented in prospective studies, with most of the evidence stemming from retrospective studies, with very similar rates in both cases (9.8% and 8.5%, respectively).

Overall, there is no clear pattern in treatment rates by study size. On the other hand, the initiation of new treatment regimen during the last month of life was mostly evaluated by studies with >2000 patients, showing lower rates compared to smaller studies.

In comparison to hospital-based studies, population-based studies generally indicated a reduced frequency of anticancer drug use in both the final 2 weeks (mean 10.0% vs. 20.1%) and 4 weeks (mean 24.5% vs. 27.5) of life. Likewise, hospital-based studies also revealed a higher rate of initiation of new treatment regime in the last month (mean 12.6%) versus population-based studies (mean 7.5%).

The frequency of anticancer drug use at end of life was similar in studies focusing on advanced cancer patients and those including all cancer stages, with mean treatment rates of 11.4% and 12.1% at 2 weeks, and 29.3% and 24.3% at 4 weeks prior to death, respectively. In contrast, the rate of introduction of a new treatment regimen at the end of life was twice as high in studies in advanced stages (14.0%) compared to those that included all cancer stages (7.4%). No major differences in rates of treatment were observed between studies including only treated patients compared to those with a mixed population.

The administration of any anticancer medication during the last 2 weeks of life was most frequently documented, with a mean rate of treatment of 11.5%. Of note, targeted therapy and immunotherapy treatment rate were only assessed at 4 weeks before deaths, with a mean rate of 26.2% and 23.2%, respectively. The initiation of a new treatment regimen was also predominantly reported for chemotherapy, with a mean rate of 8.1%.

## Discussion

Our scoping review provides an objective description about the use of anticancer drugs in the end of life of lung cancer patients. We found 68 publications with a noticeable increase in the number of publications on the topic since 2010, reaching its highest value in 2018 and maintaining a steady interest thereafter. The majority of these publications are full articles, with North America and Europe leading in the number of published works. Also, the disproportionately high number of studies originating from the Austral–Pacific region, a shift from our previous review encompassing all cancer types [21], could potentially be attributed to the high prevalence of lung cancer in Australia, as well as increasing awareness about the uncertainties related to the treatment of advanced cancer patients. [34].

The obtained results, either from a retrospective or prospective design, showed that patients with lung cancer frequently receive treatment until the very end of their life: 11.9% received anticancer drugs during the last 2 weeks and 26.1% in the last 4 weeks of their life, on average. These values were higher than some international recommendations [35].

Articles that report only the prevalence of oncologic therapies at the end-of-life period did not allow distinguishing between what are second, third, or more therapeutic lines from those that represent the first treatment administered to patients initially diagnosed in an advanced stage, which is a frequent scenario in lung cancer. That is why, to be fully informative, data disaggregated by treatment line should always be provided. Unfortunately, previous lines of treatment were infrequently reported in the studies that we identified and when that information was available in a few studies, no significant difference in median treatment lines was observed between patients who received treatment near the end of life and those who did not [36–39], or between different end-of-life time periods [28, 39, 40].

The reported studies usually lack to inform if the administered treatment at the end-of-life period corresponds to a therapeutic protocol initiated some time ago or, conversely, it represents the initiation of a new line. This latter case would be more willing to represent a potential overtreatment given the inexistence of sound evidence to expect more benefits than harms in an advanced disease when the previous treatments have failed, unless this is done in the context of an ongoing clinical trial. Furthermore, this suggests that the decision for administrating a new treatment is taken independently from past therapeutic failures.

Chemotherapy was the most administered treatment in these studies, while other therapies such as targeted therapy, immunotherapy, and biological treatment were infrequently reported. However, over the past decades, the treatment approach for lung cancer has undergone significant changes. Historically, platinum-based chemotherapy regimens were the standard treatment. However, the approval of epidermal growth factor receptor tyrosine kinase inhibitors (TKIs) heralded the era of targeted therapy [41]. More recently, immune-oncology therapies, mainly immune checkpoint inhibitors targeting the PD-1/PD-L1 pathway, have become the recommended first- and second-line treatments in Europe and North America for patients with advanced NSCLC [42, 43]. This trend is clearly demonstrated in our review, where we identified seven publications detailing the use of targeted therapy, immunotherapy, or biological treatment. All of these publications were released after 2016, with five of them published within the last 5 years. However, the former uncertainties about the positive balance between the real benefits and harms of chemotherapy probably remain with the new therapies, given the same limitations derived from the poor functional status of advanced patients who have previously underwent several therapeutic regimens. Therefore, the utilization of the new agents for treating advanced lung cancer patients should be equally cautious to avoid causing more harm than good.

Our review highlighted the lack of homogeneity in the literature when defining end-of-life periods, despite its significance as a crucial concept for the quality of provided care. Both the final 2 weeks and the final 4 weeks of life were frequently reported, indicating a lack of consensus regarding the definition of the timeframe for the end-of-life at present [24], makingthe necessary comparisons and benchmarking difficult. Our results indicate that, across all end-of-life periods, the proportion of patients receiving treatment displayed a wide range across studies, with the broadest range observed in the last 4-week period. This observation aligns with a recent review focusing on lung cancer patients, exploring the intensity of care in end-of-life settings investigating the aggressiveness of care in end-of-life care [44].

The identified variations in the proportion of patients receiving anticancer drug at the end of life suggest that cultural, societal, or healthcare system differences may influence treatment decisions [21, 45–49]. But addressing and reducing the issue of overtreatment at the end of life requires a comprehensive strategy that includes the creation and regular updating of evidence-based clinical practice guidelines by a team of diverse experts. It is important for these guidelines to be practical and to install proper mechanism to ensure adherence to them by practitioners. Moreover, involving patients in defining meaningful outcomes, prioritizing the QoL and symptom management over conventional end points such as disease progression, is also crucial. Collecting patient-centered outcome data can lead to better care customization, and sharing this data with all stakeholders can facilitate informed decision-making. Additionally, training on communication skills for healthcare providers is necessary to improve the discussion around prognoses and treatment options, ensuring that patients understand the potential benefits and risks. Utilizing decision aids can help convey this information effectively, and acknowledging uncertainties in treatment outcomes is vital. Offering emotional support during these discussions and integrating early palliative care consultations can help align care with patient preferences [50, 51].

Our review on the end-of-life anticancer drug use in lung cancer adds significant value due to the high incidence and mortality rates of the disease. Lung cancer’s rapid progression presents unique challenges in balancing life extension with quality of life, often leading to the risk of overtreatment. By focusing on lung cancer, researchers can gain more precise insights into the appropriateness and timing of treatments, particularly as the landscape of therapy has evolved rapidly with the introduction of targeted therapies and immunotherapies. This approach allows for a deeper exploration of how these newer treatments are being integrated into care plans and whether they are leading to overtreatment at the end of life. Additionally, studying lung cancer separately enables a better comparison of the outcomes of various end-of-life treatment strategies, providing more relevant findings that can directly inform clinical guidelines. Moreover, lung cancer patients have specific needs, especially related to symptom management and maintaining quality of life, which can be more effectively addressed when the research is tailored to this group.

Our findings reinforce existing guidelines from the American Society of Clinical Oncology (ASCO) [52] and the European Society for Medical Oncology (ESMO) [53] against the use of systemic anticancer therapy at the end of life, emphasizing patient-centered care and the importance of QoL. Both ASCO and ESMO advocate for early integration of palliative care, tailored use of systemic anticancer therapy based on prognosis and patient preferences, and the timely cessation of aggressive treatments that do not enhance QoL.

This scoping review has some potential limitations. Despite utilizing a high-sensitivity search strategy, there is a possibility that some relevant papers might have been overlooked. This could arise from the inherent subjectivity involved during the review stages such as study selection and narrative synthesis. However, the consistency between various reviewers throughout the references screening phase lends us confidence that the likelihood of skipped articles is minimal. Our scoping review’s fundamental goal was to gauge the prevalence of end-of-life anticancer drug application in lung cancer, as reported in comparable studies. Consequently, we sought out publications that exhibited homogeneity concerning publication dates and cancer types, disregarding other potentially significant methodological elements such as patient demographics, treatment modes, and disease stages. While adding these factors might have enhanced the consistency of the study, it could have markedly reduced the quantity of relevant publications, contradicting the review’s broad-scoping nature.

The scoping has significant strengths, such as the incorporation of the most recent articles and the detailed analysis of methodologies used in primary studies. The broad literature search yielded a substantial number of publications, and the review encompassed a diverse range of languages. For future research, it is advisable to track time trends of end-of-life anticancer drug use and gather more intricate details about treatment protocols, patient demographics, and treatment outcomes. Collecting and analyzing data on patient age, cancer type, and stage, as well as the specifics of drug regimens—dosages, schedules, and combinations—will provide a more nuanced understanding of current practices and how they align with the best interests of patients at the end of life. Furthermore, the standardization of methodologies and study environments is also recommended for producing results that can be benchmarked to advance our understanding of end-of-life anticancer treatment practices. This, in turn, will support the development of interventions aimed at optimizing care and ensuring that treatment decisions are made in the best interest of patients approaching the end of their lives.

To effectively study end-of-life cancer treatments in lung cancer, several recommendations emerge from our review. First, our findings indicate that a significant proportion of patients received oncologic treatment during the final 2–4 weeks of life, with mean rates of about 10% and 25%, respectively. This could serve as an indicator of potential overtreatment in advanced lung cancer patients, underscoring the need for careful evaluation of the benefits versus harms of continuing aggressive treatments so close to the end of life. Second, the use of newer agents, such as targeted therapy, immunotherapy, and biological treatments, in treating advanced lung cancer patients should be approached with caution. Given the potential for these treatments to cause more harm than good, their application in EOL care should be critically assessed to ensure that they genuinely benefit patients. Lastly, standardizing methodologies and study environments is crucial for producing results that can be benchmarked, thereby advancing our understanding of EOL anticancer treatment practices. This standardization will facilitate more accurate comparisons across studies and support the development of interventions that optimize care and ensure that treatment decisions align with the best interests of patients approaching the end of their lives.

## Conclusions

Our scoping review offers a comprehensive overview of the core characteristics of primary studies that aimed to explore the treatments administered during end of life in lung cancer patients. Our findings reveal a significant surge in relevant publications since 2010, with a high frequency of treatments noted across different geographic regions. The primary treatment discussed in most studies is chemotherapy, although a shift toward immune checkpoint inhibitors and TKIs for advanced NSCLC has been reported. The methodological inconsistencies across the studies present challenges in assessing and comparing the degree of end-of-life overtreatment and the relevance of its determinants. Our analysis reveals that a substantial proportion of patients receive treatment during the end-of-life phase, implying a trend toward overtreatment. In cases of lung cancer with a grim prognosis, the prevalent use of anticancer drugs calls for additional research. It is essential to standardize research methods to facilitate thorough quality evaluations and enable effective benchmarking in end-of-life care.

## Data Availability

Not applicable.

## Appendix 1: Medline search strategy

(“last day”[tiab] OR “last days”[tiab] OR “last month*”[tiab] OR “until death”[tiab] OR “near death”[tiab] OR “life end”[tiab] OR aggressive*[ti] OR terminal[ti] OR “palliative chemotherapy”[tiab] OR “end of life”[tiab] OR ((day[tiab] OR days[tiab] OR week*[tiab] OR month*[tiab]) AND (life[tiab] OR death[tiab])) OR “end stage”[ti] OR “late stage”[ti] OR “end life”[tiab]).

AND (“ctx”[ti] OR “antineoplastic”[tiab] OR “anti cancer”[ti] OR “anticancer”[ti] OR immunotherap*[ti] OR (cancer[ti] OR malignan*[ti] OR sclc[ti] OR nsclc[ti] OR neoplasm*[ti] OR tumour*[ti] OR tumor*[ti] OR metasta*[ti] AND (care[ti] OR drug*[ti] OR treatment*[ti] OR therap*[ti] OR hormone*[ti] OR immunotherapy*[ti] OR biologic*[ti] OR target*[ti]))).

AND (“lung”[MeSH Terms] OR “lung”[All Fields] OR (“bronchial”[All Fields] OR “bronchiale”[All Fields] OR “bronchials”[All Fields] OR “Bronchogenic”[All Fields] OR “mesothelioma”[MeSH Terms] OR “mesothelioma”[All Fields] OR “mesotheliomas”[All Fields] OR “mesothelioma, malignant”[MeSH Terms] OR “mesothelioma”[All Fields] AND “malignant”[All Fields]) OR “malignant mesothelioma”[All Fields] OR “lung”[MeSH Terms] OR “lung”[All Fields] OR “pulmonary”[All Fields] OR “thoracal”[All Fields] OR “thoracical”[All Fields] OR “thorax”[MeSH Terms] OR “thorax”[All Fields] OR “thoracic”[All Fields] OR “thoracics”[All Fields]).

## Appendix 2: References used for data extraction in the scoping review

1. Allen MJ, Dunn N, Guan T, Harrington J, Walpole E. End-of-life intravenous chemotherapy administration patterns in the treatment of Queensland lung and pancreas cancer patients: a 10-year retrospective analysis. Intern Med J. 2022;52(4):623–32.
2. Awano N, Izumo T, Inomata M, Kuse N, Tone M, Takada K, et al. Medical costs of Japanese lung cancer patients during end-of-life care. Jpn J Clin Oncol. 2021;51(5):769–77
3. Barbera L, Paszat L, Qiu F. End-of-Life Care in Lung Cancer Patients in Ontario: Aggressiveness of Care in the Population and a Description of Hospital Admissions. Journal of Pain and Symptom Management. 2008;35(3):267–74.
4. Bradley CJ, Yabroff KR, Mariotto AB, Zeruto C, Tran Q, Warren JL. Antineoplastic treatment of advanced-stage non-small-cell lung cancer: Treatment, survival, and spending (2000–2011). Journal of Clinical Oncology. 2017;35(5):529–35.
5. Braga S, Miranda A, Fonseca R, Passos-Coelho JL, Fernandes A, Costa JD, et al. The aggressiveness of cancer care in the last 3 months of life: A retrospective single centre analysis. Psycho-Oncology. 2007;16(9):863–8.
6. Bylicki O, Rivière F, Tournier C, Canoui-Poitrine F, Grassin F, Margery J, et al. Factors Associated With Aggressiveness of End-of-Life Care for Lung Cancer Patients and Associated Costs of Care. Clinical Lung Cancer. 2020.
7. Chan K. Aggressiveness of cancer-care in lung cancer patients near the end-of-life in an oncology center in Hong Kong. Journal of Pain Management. 2012;5(1):71–82.
8. Chan T, Ewachiw B, Huang P, Frendak L, Waldfogel J, Burdalski C, et al. Prescribing Patterns of Physicians and Financial Implications for Lung Cancer Treatment at the End of Life. Journal of Thoracic Oncology. 2018;13(10 Supplement):S818-S9.
9. Chanprasertpinyo W, Semsarn S, Tangsujaritvijit V, Ngamphaiboon N, Reungwetwattana T, Chaiviboontham S, et al. Effect of early palliative care on aggressiveness of cancer care near end of life in lung cancer patient. Journal of Thoracic Oncology. 2017;12 (11 Supplement 2):S1774-S5.
10. Chiang JK, Kao YH, Lai NS. The Impact of hospice care on survival and healthcare costs for patients with lung cancer: A national longitudinal population-based study in Taiwan. PLoS ONE. 2015;10 (9) (no pagination)(e0138773).
11. Datta S, Kelly V, Maguire J. Palliative chemotherapy with fractionated carboplatin and vinorelbine for elderly and poor performance status patients with NSCLC. Lung Cancer. 2013;1:S13-S.
12. Davidoff AJ, Canavan ME, Prsic E, Saphire M, Wang SY, Presley CJ. End-of-life patterns of symptom management and cancer-directed care among Medicare beneficiaries with lung cancer: a claims-based analysis. Support Care Cancer. 2021;29(7):3921–32.
13. De Giglio A, Tassinari E, Zappi A, Di Federico A, Lenzi B, Sperandi F, et al. The Palliative Prognostic (PaP) Score without Clinical Evaluation Predicts Early Mortality among Advanced NSCLC Patients Treated with Immunotherapy. Cancers (Basel). 2022;14(23).
14. de Man Y, Atsma F, Oosterveld-Vlug MG, Brom L, Onwuteaka-Philipsen BD, Westert GP, et al. The Intensity of Hospital Care Utilization by Dutch Patients With Lung or Colorectal Cancer in their Final Months of Life. 2019.
15. Douma G, Fransen HP, Venmans BJW, Aarts MJ. End of life treatment of metastatic lung cancer patients in the Netherlands. European Respiratory Journal Conference: European Respiratory Society International Congress, ERS. 2017;50(Supplement 61).
16. Ersek M, Miller SC, Wagner TH, Thorpe JM, Smith D, Levy CR, et al. Association between aggressive care and bereaved families’ evaluation of end-of-life care for veterans with non-small cell lung cancer who died in Veterans Affairs facilities. Cancer. 2017;123(16):3186–94.
17. Falchook AD, Dusetzina SB, Tian F, Basak R, Selvam N, Chen RC. Aggressive End-of-Life Care for Metastatic Cancer Patients Younger Than Age 65 Years. Journal of the National Cancer Institute. 2017;109(9).
18. Fujisawa D, Temel JS, Traeger L, Greer JA, Lennes IT, Mimura M, et al. Psychological factors at early stage of treatment as predictors of receiving chemotherapy at the end of life. Psycho-Oncology. 2015;24(12):1731–7.
19. Geerse OP, Hoekstra-Weebers JEHM, Stokroos MH, Burgerhof JGM, Groen HJM, Kerstjens HAM, et al. Structural distress screening and supportive care for patients with lung cancer on systemic therapy: A randomised controlled trial. European Journal of Cancer. 2017;72:37–45.
20. Gibson A, Li H, D’Silva A, Tudor R, Elegbede A, Otsuka S, et al. Factors Associated with Early Mortality in Non-Small Cell Lung Cancer Patients Following Systemic Anti-Cancer Treatment. Journal of Thoracic Oncology. 2018;13 (10 Supplement):S406-S7.
21. Goldie CL, Nguyen P, Robinson AG, Goldie CE, Kircher CE, Hanna TP. Quality of End-of-Life Care for People with Advanced Non-Small Cell Lung Cancer in Ontario: A Population-Based Study. Current Oncology. 2021;28(5):3297–315.
22. Green JB, Shapiro MF, Ettner S, Malin J, Wong MD. Chemotherapy use in lung cancer at the end of life: Predictors and variation in use. Journal of General Internal Medicine. 2011;1:S94-S5.
23. Green JB, Shapiro MF, Ettner SL, Malin J, Ang A, Wong MD. Physician variation in lung cancer treatment at the end of life. The American journal of managed care. 2017;23(4):216–23.
24. Hammerman A, Greenberg-Dotan S, Battat E, Bitterman H, Ariad S. Chemotherapy use in lung cancer patients during last 3 months of life: Current practice in a large Israeli health care organization. Journal of Clinical Oncology Conference. 2014;32(15 SUPPL. 1).
25. Hoberg J, Alt-Epping B, Freier W, Griesinger F, Strasser F, Nauck F. Do oncology and palliative care institutions respond differently to the needs of patients with lung cancer in their last month of life? Preliminary results from a multicenter survey. Palliative Medicine. 2010;1:S100-S.
26. Jessop S, Sillah T, Jeffs Y, Aslam S, Bulusu V. 30 Day mortality following systemic anti-cancer treatment (SACT) in lung cancer: Experience from a cancer unit. Lung Cancer. 2016;91 (Supplement 1):S30-S.
27. Kao SC, Van Zandwijk N, Corte P, Clarke C, Clarke S, Vardy J. Use of cancer therapy at the end of life in patients with malignant pleural mesothelioma. Supportive Care in Cancer. 2013;21(7):1879–84.
28. Kao SCH, Clarke S, Clarke C, Corte P, Van Zandwijk N, Vardy J. End of life care for malignant pleural mesothelioma (MPM) patients in Australia. Asia–Pacific Journal of Clinical Oncology 2011;4:136.
29. Karanth S, Rajan SS, Sharma G, Yamal JM, Morgan RO. Racial-Ethnic Disparities in End-of-Life Care Quality among Lung Cancer Patients: A SEER-Medicare-Based Study. Journal of Thoracic Oncology. 2018;13(8):1083–93.
30. Keating NL, Cleveland JLF, Wright AA, Brooks GA, Meneades L, Riedel L, et al. Evaluation of Reliability and Correlations of Quality Measures in Cancer Care. JAMA Netw Open. 2021;4(3):e212474.
31. King JD, Retseck J, Eickhoff JC, Hoang T, Traynor AM, Campbell TC. Integrated oncopalliative care versus standard care for patients with metastatic lung cancer: A single institution retrospective review. Journal of Clinical Oncology Conference. 2013;31(15 SUPPL. 1).
32. Kok PS, Chan H, Chao C, Descallar J, Bray V, Tognela A, et al. Timing of palliative care referral and its impact on receiving aggressive end of life care in patients with metastatic non-small cell lung cancer (NSCLC) in Southwest Sydney. Annals of Oncology. 2015;9:ix111-ix.
33. Lammers A, Slatore CG, Fromme EK, Vranas KC, Sullivan DR. Association of Early Palliative Care With Chemotherapy Intensity in Patients With Advanced Stage Lung Cancer: A National Cohort Study. 2019.
34. Lopes FC, Guerra N, Machado M, Gloria I, Brilhante M, Carvalho C, et al. Why do we treat lung cancer patients with chemotherapy until the end of life? Journal of Thoracic Oncology. 2013;2:S1006-S7.
35. Lycan TW, Jr., Buckenheimer A, Ruiz J, Russell G, Dothard AS, Ahmed T, et al. Team-Based Hospice Referrals: A Potential Quality Metric for Lung Cancer in the Immunotherapy Era. Am J Hosp Palliat Care. 2023;40(1):10–7.
36. McDermott CL, Bansal A, Ramsey SD, Lyman GH, Sullivan SD. Depression and Health Care Utilization at End of Life Among Older Adults With Advanced Non-Small-Cell Lung Cancer. Journal of Pain and Symptom Management. 2018;56(5):699–708.e1.
37. Mercado FMR, Luhrs C, Beal A, Langdon M, Secrest J, Talbot SM. Integration of palliative care services into standard oncology practice at diagnosis of metastatic lung cancer at VA New York Harbor Healthcare System. Journal of Clinical Oncology Conference. 2015;33(29 SUPPL. 1).
38. Mieras A, Becker-Commissaris A, Pasman HRW, Dingemans AMMC, Kok EV, Cornelissen R, et al. Chemotherapy and Tyrosine Kinase Inhibitors in the last month of life in patients with metastatic lung cancer: A patient file study in the Netherlands. European journal of cancer care. 2020;29(2):e13210.
39. Murillo JR, Jr., Koeller J. Chemotherapy given near the end of life by community oncologists for advanced non-small cell lung cancer. Oncologist. 2006;11(10):1095–9.
40. Nappa U, Lindqvist O, Rasmussen BH, Axelsson B. Palliative chemotherapy during the last month of life. Annals of Oncology. 2011;22(11):2375–80.
41. Nieder C, Aanes SG, Haukland EC. Days at home in the last 3 months of life: patterns-of-care analysis in patients with non-small cell lung cancer. Contemporary oncology (Poznan, Poland). 2023;27(1):41–6.
42. Nieder C, Norum J. Early palliative care in patients with metastatic non-small cell lung cancer. Ann Palliat Med. 2012;1(1):84–6.
43. Nieder C, Tollali T, Dalhaug A, Haukland E, Aandahl G, Pawinski A, et al. Active anticancer treatment during the final month of life in patients with non-small cell lung cancer. 2014.
44. Nieder C, Tollali T, Haukland E, Reigstad A, Flatoy LR, Engljahringer K. Impact of early palliative interventions on the outcomes of care for patients with non-small cell lung cancer. Supportive Care in Cancer. 2016;24(10):4385–91.
45. Oselin K, Pisarev H, Ilau K, Kiivet RA. Intensity of end-of-life health care and mortality after systemic anti-cancer treatment in patients with advanced lung cancer. BMC Cancer. 2021;21(1):274.
46. Parekh HD, Tullio K, Elson P, Davis MP, Velcheti V, Stevenson J, et al. The effect of routine early palliative care (PC) consultation on aggressiveness of care at the end of life (EOL) in patients with advanced non-small cell lung cancer (NSCLC). 2016.
47. Park M, Song I. Medical care costs of cancer in the last year of life using national health insurance data in Korea. PLoS One. 2018;13(6):e0197891.
48. Petrillo LA, El-Jawahri A, Nipp RD, Lichtenstein MRL, Durbin SM, Reynolds KL, et al. Performance status and end-of-life care among adults with non-small cell lung cancer receiving immune checkpoint inhibitors. Cancer. 2020;126(10):2288–95.
49. Philip J, Collins A, Burchell JL, Mileshkin L, Le B, Hudson P, et al. The quality of end of life care of patients with metastatic small cell lung cancer: Does it differ from other lung cancer patients? 2016.
50. Philip J, Hudson P, Bostanci A, Street A, Horey DE, Aranda S, et al. Metastatic non-small cell lung cancer: A benchmark for quality end-of-life cancer care? Medical Journal of Australia. 2015;202(3):139–44.
51. Pirl WF, Greer JA, Irwin K, Lennes IT, Jackson VA, Park ER, et al. Processes of discontinuing chemotherapy for metastatic non-small cell lung cancer at the end of life. 2014.
52. Pitson G, Matheson L, Eastman P, Rogers M. MA19.11 Population Based Analysis of End of Life Treatment Patterns in Thoracic Malignancies. Journal of Thoracic Oncology. 2019;14(10):S329-S30.
53. Saito AM, Landrum M, Neville BA, Ayanian JZ, Earle CC. The effect on survival of continuing chemotherapy to near death. BMC Palliative Care. 2011;10 (no pagination)(14).
54. Santana-Davila R, Kelley MJ, Williams CD, Eaton K, Whittle JC. Chemotherapy at the end of life (EOL) for patients with lung cancer within the VA system. 2015.
55. Sharma G, Wang Y, Graham JE, Kuo YF, Goodwin JS. Provider Continuity Prior to the Diagnosis of Advanced Lung Cancer and End-of-Life Care. PLoS ONE. 2013;8 (9) (no pagination)(e74690).
56. Smith CEP, Kamal AH, Kluger M, Coke P, Kelley MJ. National Trends in End-of-Life Care for Veterans With Advanced Cancer in the Veterans Health Administration: 2009–2016. J Oncol Pract. 2019;15(6):e568-e75.
57. Sridharan K, Paul E, Stirling RG, Li C. Impacts of multidisciplinary meeting case discussion on palliative care referral and end-of-life care in lung cancer: a retrospective observational study. Intern Med J. 2021;51(9):1450–6.
58. Stavas MJ, Martin SF, Phillips SE, Perkins SM, Shinohara ET. The utilization of chemotherapy and radiation at the end of life in individuals with metastatic non-small cell lung cancer. 2015.
59. Tanguy-Melac A, Denis P, Pestel L, Fagot-Campagna A, Gastaldi-Ménager C, Tuppin P. Intensity of care, expenditure, place and cause of death people with lung cancer in the year before their death: A French population based study. Bulletin du Cancer. 2020;107(3):308–21.
60. Thomas SP, Rice MA, T.-T AH, Heard B, Harper J, Fishkin PAS. Evaluation of chemotherapy within last 2 weeks of life: Patterns of care. Journal of Clinical Oncology Conference. 2013;31(15 SUPPL. 1).
61. Tsai HY, Chung KP, Kuo RNC. Impact of Targeted Therapy on the Quality of End-of-Life Care for Patients With Non-Small-Cell Lung Cancer: A Population-Based Study in Taiwan. Journal of Pain and Symptom Management. 2018;55(3):798–807.e4.
62. Verma V, Brian Butler E, Teh BS, Haque W. Patterns of End-of-Life Oncologic Care for Stage IV Non-small Cell Lung Cancer in the United States. Anticancer Research. 2019;39(6):3137–40.
63. Vesteghem C, Brondum RF, Mouritzen MT, Christensen HS, Bogsted M, Falkmer UG, et al. Thirty-Day Mortality Following Systemic Anticancer Therapy: Evaluating Risk Factors Without Selection Bias in a Real-World, Population-Based Cohort From 2009 to 2019. Clin Oncol (R Coll Radiol). 2022;34(8):487–96.
64. Walter J, Tufman A, Holle R, Schwarzkopf L. Comparison of costs and care of lung cancer patient at the end-of-life in germany depending on the time of survival after diagnosis. Value in Health. 2017;20 (9):A511-A.
65. Warren JL, Barbera L, Bremner KE, Yabroff KR, Hoch JS, Barrett MJ, et al. End-of-life care for lung cancer patients in the United States and Ontario. Journal of the National Cancer Institute. 2011;103(11):853–62.
66. Yang D, Qiu M, Zou LQ, Zhang W, Jiang Y, Zhang DY, et al. The role of palliative chemotherapy for terminally ill patients with advanced NSCLC. Thoracic Cancer. 2013;4(2):153–60.
67. Yoo SH, Keam B, Kim M, Kim TM, Kim DW, Heo DS. The effect of hospice consultation on aggressive treatment of lung cancer. Cancer Research and Treatment. 2018;50(3):720–8.
68. Zhu Y, Tang K, Zhao F, Zang Y, Wang X, Li Z, et al. End-of-life chemotherapy is associated with poor survival and aggressive care in patients with small cell lung cancer. Journal of Cancer Research and Clinical Oncology. 2018;144(8):1591–9.
